# One-year follow-up healthcare costs of patients diagnosed with skin cancer in Germany: a claims data analysis

**DOI:** 10.1186/s12913-022-08141-9

**Published:** 2022-06-11

**Authors:** Christian Speckemeier, Kathrin Pahmeier, Pietro Trocchi, Katrin Schuldt, Hildegard Lax, Michael Nonnemacher, Patrik Dröge, Andreas Stang, Jürgen Wasem, Silke Neusser

**Affiliations:** 1 Institute for Healthcare Management and Research, University of Duisburg-Essen, Thea-Leymann-Str. 9, 45127 Essen, Germany; 2 Institute of Medical Informatics, Biometry and Epidemiology, University Hospital of Essen, Hufelandstr. 55, 45147 Essen, Germany; 3 AOK Research Institute (WIdO), Rosenthaler Str. 31, 10178 Berlin, Germany; 4 Department of Epidemiology, Boston University School of Public Health, 715 Albany St, Boston, MA 02118 USA

**Keywords:** Cutaneous melanoma, Non-melanoma skin cancer, Skin cancer, Costs, Screening

## Abstract

**Background:**

Routine skin cancer screening (SCS) is covered by the German statutory health insurance (SHI) since 2008. The objective of this study was to compare direct healthcare costs between patients in whom skin cancer was detected by routine SCS and patients in whom skin cancer was not detected by routine SCS.

**Methods:**

A retrospective observational study of administrative claims data from a large German SHI was performed. Patients with a diagnosis of malignant melanoma (MM) or non-melanoma skin cancer (NMSC) diagnosed in 2014 or 2015 were included. Costs were obtained for one year before and one year after diagnosis and analyzed in a difference-in-differences approach using regression models. Frequency matching was applied and risk adjustment was performed. Additional analyses were conducted, separately for specific age groups, excluding persons who died during the observation period and without taking costs for screening into consideration.

**Results:**

A total of 131,801 patients were included, of whom 13,633 (10.3%) had a diagnosis of MM and 118,168 (89.7%) had a diagnosis of NMSC. The description of total costs (without risk adjustment) shows lower mean total costs among patients whose skin cancer was detected via routine SCS compared to patients in whom skin cancer was not detected by routine SCS (MM: €5,326 (95% confidence interval (CI) €5,073; €5,579) vs. €9,038 (95% CI €8,629; €9,448); NMSC: €4,660 (95% CI €4,573; €4,745) vs. €5,890 (95% CI €5,813; €5,967)). Results of the regression analysis show cost savings of 18.8% (95% CI -23.1; -8.4) through routine SCS for patients with a diagnosis of MM. These cost savings in MM patients were more pronounced in patients younger than 65 years of age. For patients with a diagnosis of NMSC, the analysis yields a non-substantial increase in costs (2.5% (95% CI -0.1; 5.2)).

**Conclusion:**

Cost savings were detected for persons with an MM diagnosed by routine SCS. However, the study could not detect lower costs due to routine SCS in the large fraction of persons with a diagnosis of NMSC. These results offer important insights into the cost structure of the routine SCS and provide opportunities for refinements.

**Supplementary Information:**

The online version contains supplementary material available at 10.1186/s12913-022-08141-9.

## Introduction

Skin cancer includes malignant melanoma (MM) and non-melanoma skin cancer (NMSC). MM is the most aggressive form of skin cancer [1]. It affects all age groups and usually causes death if diagnosed at a late disease stage [2]. The thickness of the primary tumor reflects the disease stage and is the most important prognostic factor. Therefore, early diagnosis is critical to the survival of MM patients [3]. Basal cell carcinoma (BCC) and squamous cell carcinoma (SCC) are the most common types of NMSC. Approximately ¾ of NMSCs are BCCs, which rarely metastasize and are therefore hardly ever life-threatening [4]. Prevalence is influenced by regional UV radiation, sunshine hours and sociodemographic factors [5].

In Germany, around 22,890 persons were diagnosed with MM in 2018, while 199,400 persons were diagnosed with NMSC [6]. For MM, age-standardized incidence rates have increased since the early 1970s. More than ten times as many individuals are being diagnosed with MM nowadays compared to 40 years ago [7]. A marked short-term increase in NMSC occurrence could be observed in recent years, which can partly be explained by the introduction of a nationwide routine skin cancer screening (SCS) program [8, 9]. A precursory pilot program was developed from 1999 onwards in the federal state of Schleswig–Holstein. Based on an observed decrease in MM mortality, the nationwide routine SCS program was introduced in July 2008. Germany is the first country in the world that offers a mass screening program for skin cancer [10, 11]. Aim of the implementation was to increase early detection of MM and NMSC [12]. The routine SCS enables individuals insured by the statutory health insurance (SHI) aged 35 years or more to undergo a biennial screening for skin cancer. The screening can be performed by both general practitioners and dermatologists after certification in the field of skin cancer. In case an abnormal result is found by a general practitioner, a referral to a dermatologist must be made for further clarification [12].

According to the principle of economic efficiency (Wirtschaftlichkeitsgebot) which is anchored in § 12 Social Code Book V (Sozialgesetzbuch V), health insurance services in Germany have to be sufficient, expedient and cost-effective; they may not go beyond what is necessary. Accordingly, the corresponding guideline (Krebsfrüherkennungs-Richtlinie) of the Joint Federal Committee (Gemeinsamer Bundesausschuss) stipulates regular evaluations of the quality and goal attainment of the routine SCS [12].

A number of studies have analyzed the effectiveness of routine SCS in Germany. While results concerning the reduction of MM mortality are heterogeneous, they indicate a presumable increase in the detection of skin cancer in earlier stages [13–15]. In a recently published study, routine data of AOK PLUS insured persons with a MM diagnosis from Saxony were analyzed. Insured persons in whom skin cancer was detected via routine SCS showed better survival rates than insured persons in whom skin cancer was detected outside of the routine SCS (hazard ratio 0.62; 95% confidence interval 0.48–0.80). However, the authors suppose that the difference could be e.g. due to healthy screen bias or the detection of the disease at earlier stages [16]. Based on data of the initial pilot program from Schleswig–Holstein, Stang et al. [17] estimated the number needed to screen to avoid one MM death to be around 34,000, when a risk reduction due to screening of 50% is assumed. The authors conclude that routine SCS in Germany has not yet shown a visible positive effect at the population level [17]. Another evaluation commissioned by the Joint Federal Committee analyzed physicians’ documentation data related to routine SCS. Due to specific characteristics of the datasets, only limited assertions could be derived [18]. So far, only one research project has addressed the effects of routine SCS on health care utilization and costs in Germany. Based on claims data from a German SHI, Krensel et al. [19] have compared costs of 6,041 patients diagnosed with skin cancer via routine SCS vs. costs of 6,749 patients diagnosed with skin cancer outside of the routine SCS program. Krensel et al. took screening costs for all insured persons into account, regardless of whether they were diagnosed with skin cancer or not. While treatment costs were lower for patients in the routine SCS group, these savings were outweighed by screening costs per detected skin cancer, leading to increased costs of €872–964 per patient diagnosed by routine SCS.

In this study, we want to contribute to the emerging knowledge by analyzing direct healthcare costs for the year after diagnosis of skin cancer between patients in whom skin cancer was detected by routine SCS and patients in whom skin cancer was not detected by routine SCS based on a large cohort of statutory insured persons. Due to differences in prevalence, prognosis and course of disease, MM and NMSC will be analyzed separately. By this means, this analysis aims to provide differentiated information on the financial consequences of routine SCS. The study is part of a larger research project funded by the innovation fund (Innovationsfonds, grant number 01VSF18001).

## Methods

### Study design and cohort

A retrospective observational study was performed. Nationwide claims data was provided in a anonymized form by the AOK Research Institute (Wissenschaftliches Institut der AOK, WIdO). The AOK is a large SHI provider covering about 27 million people in Germany, which represents around one third of the German population.

A sample of 450,000 AOK-insured persons diagnosed with skin cancer was provided by WIdO. Due to data restrictions, these insureds were randomly drawn from a total number of 586,475 AOK insured persons diagnosed with skin cancer in 2014/15. Of these, patients with an initial inpatient or outpatient diagnosis of skin cancer in the years 2014 or 2015 were included. Initial diagnosis defines patients who did not have a suspected or confirmed diagnosis of skin cancer in the 24 months before diagnosis. Individuals with an age between 35 (lower age limit for routine SCS) and 100 years were included if they were insured by the AOK for at least five years. Furthermore, a tumor-associated surgical treatment and/or a tumor-associated medical treatment had to be performed in the twelve months before or the six months after diagnosis of skin cancer. A tumor-associated surgical treatment was defined if the patient received at least one surgical treatment from a list of pre-defined treatments. A tumor-associated medical treatment was defined if the patient received one or more prescriptions according to the pharmaceutical registration number (Pharmazentralnummer, PZN) or the Anatomical Therapeutic Chemical (ATC) code from a list of pre-defined prescriptions. A complete list of included codes will be published elsewhere. Persons with diagnosis of MM (International Classification of Diseases (ICD) code C43) and NMSC (ICD code C44) were assessed separately. Persons who have been identified with both MM and NMSC were assigned to the cohort of the skin cancer which was diagnosed first. The date of diagnosis was defined as the date of discharge (inpatient and outpatient hospital care) or the date of service provision (outpatient care). Persons were assigned to the screening group if a routine SCS according to fee schedule item (Gebührenordnungsziffer) 01745 or 01746 was performed in the three months prior to diagnosis of skin cancer. Thus, patients were assigned to four different groups, depending on tumor entity (C43; C44) and the pattern of tumor detection.

### Resource utilization and cost analyses

A calculation of the total costs in the year before and after diagnosis of skin cancer was carried out. The charges considered in the analysis comprise data on direct costs contained in the claims data in six areas, namely (i) inpatient hospitalization costs, (ii) outpatient hospitalization costs, (iii) costs for pharmaceuticals, (iv) outpatient healthcare costs, (v) remedy costs and (vi) costs for rehabilitation paid by the health insurance. Analysis was performed from the SHI perspective and thus, net costs without copayments were considered. Total costs in these six areas were analyzed. For pharmaceutical costs, the date of submission by the pharmacy was considered and for remedy costs, the invoice date was considered instead of prescription date. Due to the structure of the claims data, inpatient hospitalization costs, outpatient healthcare costs and costs for rehabilitation were only available for time periods (i.e., timespan from beginning to end of treatment). Only costs that occurred in the relevant observation period were included (on a pro-rata basis). For example, in cases that fell in both the pre- and post-observation periods, the costs were allocated proportionally over the days in the respective period. Differences between groups were tested for significance according to Mann–Whitney-U-test.

### Statistical analysis

A regression analysis was undertaken to predict the cost differences of patients who underwent routine SCS compared to patients diagnosed outside of the routine SCS. Statistical analysis was performed with SAS V9.4 (SAS Institute Inc., Cary, NC, USA). A difference-in-differences (DiD) approach was applied to control for possible background changes in outcomes that occur with time. For the DiD estimation, a pre-observation period of one year was defined, which ended one day before the date of skin cancer diagnosis. A generalized linear model with log link and gamma distribution was fitted to the data by means of maximum likelihood estimation and by estimating the parameters of the model via an iterative fitting process. The distribution was determined based on a modification of the Park’s test for heteroscedasticity proposed by Manning & Mullahy [20]. For the group of persons with an MM diagnosis, the test resulted in a parameter estimate of 1.89 (1.86–1.92) and for the group of persons with an NMSC diagnosis, the parameter estimate was 1.84 (1.83–1.85). As parameter estimates close to 2 indicate a gamma distribution, this distribution was used.

Frequency matching was applied. Included persons were matched by diagnosis (C43, C44), five-year age group, sex, and federal state of residence. To control for possible differences between the two groups and limit the potential for bias, the regression analysis adjusted for the Elixhauser Comorbidity Index and Pharmacy-based Metric. The Elixhauser Comorbidity Index is a risk adjustment measure based on distinctive ICD codes [21], which was adapted to the 10^th^ Revision of ICD (ICD-10) by Quan et al. [22]. The Pharmacy-based Metric was developed to explain variation in healthcare utilization based on the ATC classification [23]. Both Elixhauser Comorbidity Index and Pharmacy-based Metric were calculated for the pre-observation period. The resulting regression equation for individual i at time t is

$${\mathrm{Total\ costs}}_{\mathrm{it}}={\upbeta }_{0}+{\upbeta }_{1}*{\mathrm{Scr}}_{\mathrm{i}}+{\upbeta }_{2}*{\mathrm{Per}}_{\mathrm{it}}+{\upbeta }_{3}*\left({\mathrm{Scr}}_{\mathrm{i}}*{\mathrm{Per}}_{\mathrm{it}}\right)+{\upbeta }_{4}*{\mathrm{PBM}}_{\mathrm{i}}+{\upbeta }_{5}*{\mathrm{EH}}_{\mathrm{i}}+{\upvarepsilon }_{\mathrm{it}}$$

where.

- Scr = dummy-variable to distinguish between routine SCS that has taken place (1) and no routine SCS (0)
- Per = dummy-variable to distinguish between pre- (0) and post-observation (1) period
- PBM = Pharmacy-based Metric
- EH = Elixhauser Comorbidity Index

and coefficient estimator ${\upbeta }_{3}$ is of main interest as a DiD estimator [24]:

$$\mathrm{DiD }= \left({\mathrm{costs}}_{\mathrm{treatment},\mathrm{ post}}-{\mathrm{costs}}_{\mathrm{control},\mathrm{ post}}\right)-({\mathrm{costs}}_{\mathrm{treatment},\mathrm{ pre}}-{\mathrm{costs}}_{\mathrm{control},\mathrm{ pre}})$$

Additional analyses were conducted in which (i) persons who died in the observation period were excluded, (ii) only persons with an age of 35 to 64 years were included, (iii) only persons from 65 years of age onwards were included, and (iv) where screening costs for the individual persons diagnosed via routine SCS were subtracted.

## Results

### Included population

The selection of the included study population will be reported in detail elsewhere. Patients without a relevant diagnosis of skin cancer and patients with a diagnosis of skin cancer before 2014 were excluded (*n* = 271,613). A total of 7,474 patients were excluded because they were younger than 35 or older than 100 years of age. Further, 6,466 patients were excluded because information on federal state was missing or because they have been AOK-insured for less than five years. Finally, 32,646 patients have been excluded because no tumor-associated treatment was performed.

The inclusion criteria were met by 13,633 persons with a diagnosis of MM, of which 6,480 (47.5%) underwent routine SCS. In addition, a total of 118,168 persons with a diagnosis of NMSC have been included, of which 43,308 (36.6%) persons underwent routine SCS. Patient characteristics including scores of the risk adjustment measures are shown in Table 1. Persons with a diagnosis of MM were, on average, five years younger in the routine SCS group when compared to the control group. Persons with a diagnosis of NMSC detected via routine SCS were 2.1 years younger, on average. For persons with a diagnosis of MM and NMSC, scores of Elixhauser Comorbidity Index and Pharmacy-based Metric were lower in the routine SCS group compared to the control group, respectively. An overview of the results for the single scores of Elixhauser Comorbidity Index and Pharmacy-based metric is shown in Supplementary Table 1, Additional File 1 and Supplementary Table 2, Additional File 2.

**Table 1:** Characteristics of the included population

	**MM**			**NMSC**		
	**Routine SCS**	**Control**	**Total**	**Routine SCS**	**Control**	**Total**
n	6480	7153	13,633	43,308	74,860	118,168
Female sex, %	49.4*	54.4*	52.0	51.4*	53.5*	52.7
Age: mean (median)	63.3* (64.4)	66.2* (67.9)	64.8 (66.1)	70.8* (73.7)	74.0* (76.2)	72.8 (75.3)
Elixhauser Comorbidity Index, pre-observation period						
Mean	3.14*	3.51*	3.34	3.74*	4.13*	3.99
Median	3	3	3	3	4	4
SD	2.63	2.74	2.69	2.70	2.78	2.76
Min	0	0	0	0	0	0
Max	16	19	19	18	18	18
Pharmacy-based Metric, pre-observation period						
Mean	3.20*	3.55*	3.39	3.69*	4.07*	3.93
Median	3	3	3	3	4	4
SD	2.36	2.47	2.42	2.39	2.47	2.45
Min	0	0	0	0	0	0
Max	14	14	14	16	17	17

*SCS* skin cancer screening, *SD* standard deviation

^*^significant difference according to Mann–Whitney-U-test between routine SCS and control, *p* < 0.05

### Treatment costs

Descriptive statistics of costs for inpatient hospitalization, outpatient hospitalizations, pharmaceuticals, outpatient healthcare, remedies and rehabilitation as well as total costs as a sum of these components for the post-observation period are shown in Table 2. Respective costs for the pre-observation period are shown in Supplementary Table 3, Additional File 3. Of note, the low minimum values occur due to the fact that costs for inpatient hospitalizations, outpatient healthcare and rehabilitation were calculated on a pro rata basis. For both indications, average costs in the respective six areas and average total costs were lower in the pre-observation as well as in the post-observation period in the group of persons with routine SCS compared to the control group.

**Table 2:** Treatment costs in the post-observation period

	**MM**			**NMSC**		
	**Routine SCS**	**Control**	**Total**	**Routine SCS**	**Control**	**Total**
n	6480	7153	13,633	43,308	74,860	118,168
**Inpatient hospitalization costs**						
Mean	2767.10	4573.43	3714.85	2216.85	3004.00	2715.51
Median	0.00	1428.09	315.96	0.00	0.00	0.00
SD	6814.68	9890.87	8614.61	6683.39	7684.00	7342.92
Min	0.00	0.00	0.00	0.00	0.00	0.00
Max	122,713.87	312,520.45	312,520.45	228,671.61	309,797.95	309,797.95
**Outpatient hospital costs**						
Mean	67.01	107.81	88.41	33.24	41.52	38.49
Median	0.00	0.00	0.00	0.00	0.00	0.00
SD	354.06	494.66	434.02	213.64	288.76	263.76
Min	0.00	0.00	0.00	0.00	0.00	0.00
Max	18,173.77	23,034.12	23,034.12	21,251.64	32,051.07	32,051.07
**Costs for pharmaceuticals**						
Mean	1233.64	2897.47	2106.62	1040.48	1356.63	1240.76
Median	210.32	348.40	272.99	310.14	426.03	380.69
SD	5575.41	11,783.59	9397.53	4254.56	5157.71	4848.66
Min	0.00	0.00	0.00	0.00	0.00	0.00
Max	123,840.14	193,379.38	193,379.38	434,528.54	589,672.64	589,672.64
**Outpatient healthcare costs**						
Mean	1090.04	1255.63	1176.92	1173.56	1248.93	1221.30
Median	819.56	904.63	859.22	860.50	890.55	878.53
SD	1637.92	1887.42	1775.08	2147.24	2306.42	2249.67
Min	0.00	0.00	0.00	0.00	0.00	0.00
Max	33,857.41	35,322.54	35,322.54	51,681.75	85,311.71	85,311.71
**Remedy costs**						
Mean	102.73	133.49	118.87	114.16	140.43	130.80
Median	0.00	0.00	0.00	0.00	0.00	0.00
SD	322.09	411.63	372.07	359.91	452.41	421.07
Min	0.00	0.00	0.00	0.00	0.00	0.00
Max	6855.40	7845.54	7845.54	9346.20	16,780.75	16,780.75
**Costs for rehabilitation**						
Mean	65.48	70.60	68.17	81.23	98.32	92.06
Median	0.00	0.00	0.00	0.00	0.00	0.00
SD	534.11	529.33	531.60	579.65	692.11	653.19
Min	0.00	0.00	0.00	0.00	0.00	0.00
Max	16,980.56	17,139.24	17,139.24	24,232.00	38,883.30	38,883.30
**Total costs**						
Mean	5326.00	9038.42	7273.84	4659.52	5889.79	5438.50
Median	2108.40	3682.95	2886.44	1902.03	2577.06	2297.33
SD	10,399.32	17,673.30	14,788.79	9151.26	10,717.84	10,188.89
Min	2.68	1.85	1.85	1.24	0.60	0.60
Max	187,263.84	318,825.87	318,825.87	439,873.87	593,014.08	593,014.08

All costs in €. Costs for screening are included. *SCS* skin cancer screening, *SD* standard deviation

Results of the main regression analyses are shown in Table 3. The matched and adjusted DiD estimation indicates cost savings of 18.8% (95% CI -23.1; -8.4) through routine SCS for patients with a diagnosis of MM. For patients with a diagnosis of NMSC, the analysis yields an increase in costs of 2.5% (95% CI -0.1; 5.2) through routine SCS.

**Table 3:** Results of the main regression analyses

Parameter	Estimate	Standard error	95% CI		Z	Pr >|Z|
**MM, main analysis**						
Intercept	7.5284	0.0336	7.4626	7.5942	224.16	< .0001
Scr	-0.3563	0.0317	-0.4185	-0.2942	-11.24	< .0001
Per	0.8360	0.0317	0.7739	0.8981	26.39	< .0001
Scr*Per	-0.1720^a^	0.0466	-0.2634^a^	-0.0807^a^	-3.69	0.0002
EH	0.1036	0.0061	0.0916	0.1155	17.01	< .0001
PBM	0.1391	0.0082	0.1231	0.1551	17.04	< .0001
**NMSC, main analysis**						
Intercept	7.3222	0.0142	7.2943	7.3501	514.17	< .0001
Scr	-0.2106	0.0132	-0.2364	-0.1847	-15.95	< .0001
Per	0.3476	0.0085	0.3310	0.3643	40.95	< .0001
Scr*Per	0.0249^a^	0.0130	-0.0006^a^	0.0504^a^	1.92	0.0555
EH	0.1013	0.0027	0.0960	0.1065	37.95	< .0001
PBM	0.1640	0.0031	0.1579	0.1701	52.64	< .0001

*CI* Confidence interval, *Scr* dummy-variable to distinguish between routine SCS that has taken place and no routine SCS, *Per* dummy-variable to distinguish between pre- and post-observation period, *PBM* Pharmacy-based Metric, *EH* Elixhauser Comorbidity Index; Screening*Period denotes the DiD estimator

^a^Differences in coefficients between table and text arise as the text reports exact numbers, calculated as ((e^coefficient)-1)*100%

### Results of the additional analyses

Detailed results of the additional analyses are reported in Supplementary Table 4, Additional File 4. When persons who died in the post observation period were excluded, the estimation yields cost savings of 17.7% (95% CI -29.5; -7.0) through routine SCS for patients with a diagnosis of MM. For patients with a diagnosis of NMSC who underwent routine SCS, costs are 1.0% (95% CI -1.6; 3.6) higher. Including persons from 35 to 64 years of age only indicates cost savings of 39.0% (95% CI -59.3; -21.2) through routine SCS in patients with MM. In patients with NMSC, the difference between screening and control group in this age group is 2.8% (95% CI -8.5; 2.7). Including persons from 65 years of age onwards only results in reduced cost of 4.5% (95% CI -17.2; 7.4) and increased costs of 4.4% (95% CI 1.3; 7.5) through routine SCS for patients diagnosed with MM and NMSC, respectively. Finally, when subtracting the costs for screening of the individual persons undergoing routine SCS, cost savings of 18.5% (95% CI -29.8; -8.1) and increased costs of 2.8% (95% CI 0.2; 5.5) arise for patients diagnosed with MM and NMSC, respectively.

### Screening costs for all persons who underwent screening

Of the total number of 131,801 insured persons with skin cancer, 49,788 persons had undergone routine SCS. Based on the fact that the sample of 450,000 was randomly drawn from a sample of 586,475 insured persons with diagnosis of skin cancer, a total number of 64,888 AOK insured persons with positive SCS is estimated (49,788*586,475/450,000). According to the SHI frequency statistic (GKV-Frequenzstatistik), which depicts the services that have been billed for SHI-accredited healthcare services, a total of 4,900,618 routine SCS were performed in AOK insured persons in 2014 and 2015 (of which 53% were billed with EBM 01746 and 47% with EBM 01745), leading to total screening costs of €96,162,994. Applying these costs to the estimated 64,888 individuals with routine SCS would result in screening costs of €1,482 per detected skin cancer.

## Discussion

A total of 131,801 persons with skin cancer were included in this study. The majority of persons (*n*= 118,168) were diagnosed with NMSC, while 13,633 persons were diagnosed with MM. Almost half of the persons with a diagnosis of MM (47.5%; 6480/13,633) underwent routine SCS while 36.6% (43,308/118,168) of NMSC patients underwent routine SCS. These numbers are similar to the proportions seen in the precursory pilot project SCREEN, in which 50.0% (585/1169) of MM and 37.8% of NMSC (2353/6218) patients were detected via routine SCS [10].

The cost-analysis of this study regarding the healthcare costs of patients diagnosed with skin cancer in Germany shows for MM lower healthcare costs for patients with a diagnosis of MM detected via routine SCS compared to patients who were diagnosed outside of the routine SCS program. Analysis of specific age groups indicates that this difference in costs was highest in patients younger than 65 years of age. In contrast, for patients diagnosed with NMSC there seems to be a tendency towards slightly higher costs in the group of persons with NMSC detected via routine SCS compared to the control group. However, both MM and NMSC are detected by routine SCS, with the program aiming to detect early stages. Considering the high proportion of NMSC in the total number of skin cancers diagnosed, the benefit of the routine SCS remains unclear. Since only cost analyses have been carried out so far, a need for a well-conducted cost-effectiveness analysis can be derived. Up to now, only one other study has assessed economic aspects of the German routine SCS program. Krensel et al. [19] included 12,790 patients with a diagnosis of skin cancer and analyzed the costs for the year after diagnosis by applying entropy balancing, a DiD approach and generalized linear models. Despite some methodological similarities between the study of Krensel et al. and this study, a number of differences exist. For example, Krensel et al. included patients with a biopsy or excision in order to verify the diagnosis while in this study only persons who underwent tumor-associated surgical treatment and/or a tumor-associated medical treatment have been included. Another major difference is the separate analysis of patients with MM and NMSC in this study. In their aggregate analysis, Krensel et al. [19] found lower costs for patients in the routine SCS group for the observation period. However, these cost savings were outweighed by screening costs for all persons covered by the SHI, which resulted in a cost increase of €872–964 for patients diagnosed by routine SCS. In our analysis, a definite assertion about the screening costs per detected individual with skin cancer could not be made. Our sample included individuals who underwent tumor-associated surgical treatment and/or a tumor-associated medical treatment only. Due to these restrictive inclusion criteria, it cannot be ensured that all skin cancer cases were actually included in the analysis. In addition, our model specification allows us to derive percentage differences instead of absolute cost differences and consequently, no precise statement can be made about the influence of screening costs on the observed savings. Of note, our estimated screening costs of €1,482€ per detected skin cancer are slightly higher than the €1,339 to €1,431 estimated by Krensel et al. [19], which is most likely due to the restrictive inclusion criteria in our study.

Based on the results of 108,000 total skin examinations, Guther et al. [25] developed a targeted risk-group model for routine SCS with the aim to facilitate targeted screening. The model is composed of a number of risk factors, including hair color, age, and history of skin cancer. The authors postulate that using their risk calculator can reduce the number needed to screen by around 50% and offers increased sensitivity for MM detection and similar sensitivity for NMSC detection when compared to the routine SCS program. While most of the risk factors mentioned by Guther et al. were not accessible in the claims data, the analysis of specific age groups shows strongly pronounced cost differences of 39% (95% CI -59.3; 21.2) in the group of persons aged 35 to 64 years. This observation may be due to the fact that early MM stages are more likely to be detected by routine SCS in this age group than outside of the routine SCS. The earlier treatment would then lead to lower costs. Analogously, evidence shows that the introduction of routine SCS leads to an increased incidence of in situ and invasive skin cancer, accompanied by an increasing rate of thin MM and decreasing rate of thick MM [26]. However, in our study information on tumor stage was not available and consequently, differences related to tumor stage between the groups remain speculative.

The present study has several limitations. First, per definition, screening involves testing asymptomatic persons. Thus, individuals with suspected skin cancer who have participated in the SCS program must not be included in the routine SCS group. However, based on the available data, it was not possible to identify symptomatic patients in the routine SCS group who attended the SCS program to undergo diagnostic testing of their suspected skin cancer. This is due to the fact that in Germany, the medical documentation of dermatologists is anonymous and not contained in the claims data. Up to now, no data linkage is possible. This likely results in an overestimation of the true number of persons with routine SCS-detected skin cancer in the routine SCS group. This limitation was also mentioned in context of the precursory pilot program [10]. Within the scope of this study, total costs in the six areas were determined and compared for the observation period, which means that also non-tumor-related costs were included. A marked right-skewedness was seen in total costs, indicating a non-negligible influence of very expensive procedures that do not necessarily have to be skin cancer-related. Further, it cannot be ruled out that persons participating in routine SCS might have a more favorable health behavior and prevalence of comorbidities when compared to persons not attending the routine SCS program. Persons with comorbidities are known to pose a higher burden on the healthcare system. Although risk adjustment was performed by employing Elixhauser Comorbidity Index and Pharmacy-based Metric, it cannot be ruled out that certain effects remain uncontrolled. Finally, this analysis only included insureds from one SHI and therefore, the patient population might not be representative of the entire population of statutory insured persons. However, as described above, the distribution of tumor entities was comparable to the proportions seen in the pilot project SCREEN [10].

## Conclusions

Concluding, the results of this study indicate that routine SCS leads to lower costs in the year after diagnosis for persons diagnosed with MM. Cost differences were more pronounced in younger persons. The results do not provide a confirmation that routine SCS is viable from an economic perspective for persons diagnosed with NMSC. Methodological limitations inherent to the evaluated data have to be considered, due to which the screening costs of all insured persons were not taken into account. Future studies should aim to investigate the cost-effectiveness of the German routine SCS. These studies should seek to compare the specific patient benefits, such as an increase in quality of life or a reduction in the recurrence rate, with the additional costs of the routine SCS. Advancements of the German routine SCS program should address the problem that the majority of detected skin cancers do not clearly benefit from early detection. In addition, the question of whether a screening offer for specific risk populations would be an advisable alternative to the routine SCS program remains.

## Supplementary Information

**Additional file 1. **

**Additional file 2. **

**Additional file 3. **

**Additional file 4. **

## Data Availability

The claims data supporting the findings of this study are not publicly accessible. Data have been provided by the AOK Research Institute (Wissenschaftliches Institut der AOK, WIdO) for the purpose of the study only. Therefore, the datasets used and/or analysed during the current study are available from the corresponding author on reasonable request.

## Abbreviations

ATC: Anatomical Therapeutic Chemical Classification
BCC: Basal cell carcinoma
MM: Malignant melanoma
NMSC: Non-melanoma skin cancer
OPS: German procedure classification (Operationen- und Prozedurenschlüssel)
SCC: Squamous cell carcinoma
SCS: Skin cancer screening
WIdO: AOK Research Institute (Wissenschaftliches Institut der AOK)
ICD: International Classification of Diseases
DiD: Difference-in-differences
PZN: Pharmazentralnummer
SHI: Statutory Health Insurance
