# Supplementary material for: One-year follow-up healthcare costs of patients diagnosed with skin cancer in Germany: a claims data analysis
Source: BMC Health Serv Res. 2022 Jun 11;22:771. doi: 10.1186/s12913-022-08141-9 (PMC9188701; doi:10.1186/s12913-022-08141-9)
Supplement: Supplementary file 1 — Additional file 1. [file 12913_2022_8141_MOESM1_ESM.docx]

**Supplementary Table 1.** Elixhauser Comorbidity Index, mean values, pre-observation period.

|  | **C43** | | | **C44** | | |
| --- | --- | --- | --- | --- | --- | --- |
|  | **Routine SCS** | **Control** | **p-value** | **Routine SCS** | **Control** | **p-value** |
| Congestive heart failure | 0.6147 | 0.9330 | <.0001 | 0.9855 | 1.3530 | <.0001 |
| Cardiac arrhythmias | 1.4560 | 1.7526 | 0.0010 | 2.0473 | 2.4096 | <.0001 |
| Valvular disease | 0.5904 | 0.7201 | 0.0163 | 0.8593 | 1.0208 | <.0001 |
| Pulmonary circulation disorders | 0.1201 | 0.1545 | 0.1435 | 0.1653 | 0.2077 | <.0001 |
| Peripheral vascular disorders | 0.8906 | 1.1042 | 0.0067 | 1.2034 | 1.4227 | <.0001 |
| Hypertension, uncomplicated | 4.7918 | 5.1752 | 0.0027 | 5.6343 | 6.0957 | <.0001 |
| Hypertension, complicated | 0.4793 | 0.5589 | 0.0560 | 0.6524 | 0.8136 | <.0001 |
| Paralysis | 0.0952 | 0.1771 | 0.0002 | 0.1159 | 0.1926 | <.0001 |
| Other neurological disorders | 0.4860 | 0.5777 | 0.1060 | 0.5671 | 0.7391 | <.0001 |
| Chronic pulmonary disease | 1.5895 | 1.6548 | 0.4378 | 1.8256 | 1.8861 | 0.0727 |
| Diabetes, uncomplicated | 2.3944 | 2.5603 | 0.1742 | 2.7858 | 3.1688 | <.0001 |
| Diabetes, complicated | 1.1769 | 1.2873 | 0.2311 | 1.4526 | 1.8192 | <.0001 |
| Hypothyroidism | 0.7321 | 0.7797 | 0.3613 | 0.7726 | 0.7367 | 0.0806 |
| Renal failure | 0.9724 | 1.2825 | 0.0642 | 1.6400 | 2.0494 | <.0001 |
| Liver disease | 0.7526 | 0.6836 | 0.1994 | 0.7877 | 0.8192 | 0.1420 |
| Peptic ulcer disease excluding bleeding | 0.0846 | 0.0851 | 0.9642 | 0.1007 | 0.1094 | 0.1080 |
| AIDS/HIV | 0.0080 | 0.0351 | 0.1298 | 0.0244 | 0.0262 | 0.8606 |
| Lymphoma | 0.2481 | 0.3650 | 0.1587 | 0.2484 | 0.3234 | 0.0050 |
| Metastatic cancer | 0.3495 | 0.8142 | <.0001 | 0.2869 | 0.4522 | <.0001 |
| Solid tumor without metastasis | 2.0451 | 3.0650 | <.0001 | 2.4064 | 2.6757 | <.0001 |
| Rheumatoid arthritis/ collagen vascular diseases | 0.6327 | 0.6397 | 0.9223 | 0.7551 | 0.8275 | 0.0130 |
| Coagulopathy | 0.1787 | 0.1611 | 0.5620 | 0.2265 | 0.2561 | 0.0264 |
| Obesity | 0.8285 | 0.9133 | 0.1106 | 0.8291 | 0.9010 | <.0001 |
| Weight loss | 0.0437 | 0.0432 | 0.9611 | 0.0444 | 0.0714 | 0.0018 |
| Fluid and electrolyte disorders | 0.1000 | 0.1583 | 0.1714 | 0.1519 | 0.1825 | 0.0052 |
| Blood loss anemia | 0.0211 | 0.0080 | 0.0910 | 0.0164 | 0.0180 | 0.4902 |
| Deficiency anemia | 0.1627 | 0.2181 | 0.0923 | 0.2129 | 0.2375 | 0.0416 |
| Alcohol abuse | 0.1708 | 0.2170 | 0.1810 | 0.1448 | 0.1734 | 0.0026 |
| Drug abuse | 0.0321 | 0.1035 | 0.1983 | 0.0619 | 0.0703 | 0.6014 |
| Psychoses | 0.1586 | 0.2344 | 0.0693 | 0.1215 | 0.1744 | <.0001 |
| Depression | 1.8123 | 1.9054 | 0.3705 | 1.7645 | 1.8764 | 0.0019 |
