# Supplementary material for: One-year follow-up healthcare costs of patients diagnosed with skin cancer in Germany: a claims data analysis
Source: BMC Health Serv Res. 2022 Jun 11;22:771. doi: 10.1186/s12913-022-08141-9 (PMC9188701; doi:10.1186/s12913-022-08141-9)
Supplement: Supplementary file 2 — Additional file 2. [file 12913_2022_8141_MOESM2_ESM.docx]

**Supplementary Table 2.** Pharmacy-based Metrics, mean values, pre-observation period.

|  | **C43** | | | **C44** | | |
| --- | --- | --- | --- | --- | --- | --- |
|  | **Routine SCS** | **Control** | **p-value** | **Routine SCS** | **Control** | **p-value** |
| Antiplatelet | 0.0299 | 0.0319 | 0.5145 | 0.0374 | 0.0449 | <.0001 |
| Anticoagulants | 0.0847 | 0.1163 | <.0001 | 0.1219 | 0.1476 | <.0001 |
| Epilepsy | 0.0474 | 0.0589 | 0.0029 | 0.0562 | 0.0705 | <.0001 |
| Hypertension | 0.0924 | 0.1111 | 0.0003 | 0.1194 | 0.1437 | <.0001 |
| HIV | 0.0009 | 0.0017 | 0.2274 | 0.0006 | 0.0011 | 0.0043 |
| Rheumatic conditions | 0.0892 | 0.1072 | 0.0004 | 0.1039 | 0.1146 | <.0001 |
| Hyperlipidemia | 0.2313 | 0.2398 | 0.2468 | 0.2941 | 0.3098 | <.0001 |
| Malignancies | 0.0063 | 0.0159 | <.0001 | 0.0069 | 0.0122 | <.0001 |
| Parkinson's disease | 0.0196 | 0.0292 | 0.0003 | 0.0296 | 0.0367 | <.0001 |
| Renal disease | 0.0048 | 0.0069 | 0.1147 | 0.0096 | 0.0122 | <.0001 |
| End stage renal disease | 0.0032 | 0.0045 | 0.2480 | 0.0059 | 0.0078 | 0.0001 |
| Anti-arrhythmic | 0.0241 | 0.0347 | 0.0003 | 0.0439 | 0.0567 | <.0001 |
| Ischemic heart disease/Angina | 0.3951 | 0.4363 | <.0001 | 0.4994 | 0.5552 | <.0001 |
| Congestive heart failure | 0.4492 | 0.5036 | <.0001 | 0.5638 | 0.6267 | <.0001 |
| Diabetes | 0.1363 | 0.1564 | 0.0009 | 0.1604 | 0.1923 | <.0001 |
| Glaucoma | 0.0420 | 0.0439 | 0.5805 | 0.0567 | 0.0626 | <.0001 |
| Liver failure | 0.0037 | 0.0070 | 0.0091 | 0.0051 | 0.0081 | <.0001 |
| Acid peptic disease | 0.2807 | 0.3173 | <.0001 | 0.3354 | 0.3715 | <.0001 |
| Transplantation | 0.0051 | 0.0039 | 0.3034 | 0.0070 | 0.0070 | 0.9215 |
| Respiratory illness, asthma | 0.1139 | 0.1202 | 0.2505 | 0.1297 | 0.1328 | 0.1404 |
| Thyroid disorders | 0.1738 | 0.1872 | 0.0419 | 0.1968 | 0.1926 | 0.0726 |
| Gout | 0.0872 | 0.0998 | 0.0116 | 0.1133 | 0.1335 | <.0001 |
| Inflammatory bowel disease, chronic | 0.0065 | 0.0063 | 0.8891 | 0.0076 | 0.0081 | 0.2967 |
| Pain and inflammation | 0.3472 | 0.3568 | 0.2437 | 0.3531 | 0.3527 | 0.8860 |
| Pain | 0.0460 | 0.0678 | <.0001 | 0.0559 | 0.0785 | <.0001 |
| Depression | 0.1269 | 0.1346 | 0.1789 | 0.1277 | 0.1490 | <.0001 |
| Psychotic illness | 0.0273 | 0.0445 | <.0001 | 0.0318 | 0.0498 | <.0001 |
| Bipolar disorders | 0.0012 | 0.0020 | 0.2939 | 0.0017 | 0.0016 | 0.7194 |
| Anxiety and tension | 0.0341 | 0.0439 | 0.0033 | 0.0402 | 0.0497 | <.0001 |
| Hepatitis | 0.0000 | 0.0004 | 0.0992 | 0.0000 | 0.0001 | 0.4973 |
