# Supplementary material for: One-year follow-up healthcare costs of patients diagnosed with skin cancer in Germany: a claims data analysis
Source: BMC Health Serv Res. 2022 Jun 11;22:771. doi: 10.1186/s12913-022-08141-9 (PMC9188701; doi:10.1186/s12913-022-08141-9)
Supplement: Supplementary file 3 — Additional file 3. [file 12913_2022_8141_MOESM3_ESM.docx]

**Supplementary Table 3.** Treatment costs in the pre-observation period.

|  | **MM** | | | **NMSC** | | |
| --- | --- | --- | --- | --- | --- | --- |
|  | **Routine SCS** | **Control** | **Total** | **Routine SCS** | **Control** | **Total** |
| n | 6480 | 7153 | 13633 | 43308 | 74860 | 118168 |
| **Inpatient hospitalization costs** | | | | | | |
| Mean  Median  SD  Min  Max | 1236.63  0.00  3752.72  0.00  92064.16 | 2343.34  0.00  5917.70  0.00  104332.77 | 1817.30  0.00  5037.02  0.00  104311.77 | 1385.20  0.00  4538.16  0.00  209850.10 | 2069.89  0.00  5951.08  0.00  255844.82 | 1818.95  0.00  5085.65  0.00  255844.82 |
| **Outpatient hospital costs** | | | | | | |
| Pre-observation period | | | | | | |
| Mean  Median  SD  Min  Max | 30.96  0.00  194.46  0.00  7582.97 | 45.17  0.00  276.66  0.00  9900.00 | 38.41  0.00  241.21  0.00  9900.00 | 29.53  0.00  191.08  0.00  9529.26 | 32.48  0.00  191.67  0.00  13748.20 | 31.40  0.00  191.46  0.00  13748.20 |
| **Costs for pharmaceuticals** | | | | | | |
| Mean  Median  SD  Min  Max | 768.74  168.24  3174.50  0.00  90844.14 | 1085.96  250.47  4312.03  0.00  103997.02 | 935.18  204.51  3817.04  0.00  103997.02 | 891.76  278.83  3227.79  0.00  136659.17 | 1,159.11  381.97  4747.94  0.00  581162.71 | 1,061.13  340.71  4256.28  0.00  581162.71 |
| **Outpatient healthcare costs** | | | | | | |
| Mean  Median  SD  Min  Max | 953.64  717.88  1542.16  0.00  33521.34 | 1017.90  759.22  1717.60  0.00  33993.96 | 987.36  740.26  1636.81  0.00  33993.96 | 1033.46  754.19  2014.21  0.00  53358.14 | 1107.46  792.68  2202.24  0.00  87499.12 | 1080.34  777.45  2135.54  0.00  87499.12 |
| **Remedy costs** | | | | | | |
| Mean  Median  SD  Min  Max | 88.25  0.00  294.56  0  5454.87 | 113.40  0.00  400.17  0.00  9179.90 | 101.44  0.00  354.13  0.00  9179.90 | 106.87  0.00  337.52  0.00  10136.72 | 130.15  0.00  422.53  0.00  15200.97 | 121.62  0.00  393.67  0.00  15200.97 |
| **Costs for rehabilitation** | | | | | | |
| Mean  Median  SD  Min  Max | 49.41  0.00  477.08  0.00  16800.92 | 77.00  0.00  544.41  0.00  10992.66 | 63.88  0.00  513.68  0.00  16800.92 | 72.60  0.00  531.27  0.00  20850.60 | 90.11  0.00  625.23  0.00  26415.36 | 83.69  0.00  592.58  0.00  26415.36 |
| **Total costs** | | | | | | |
| Mean  Median  SD  Min  Max | 3127.63  1274.92  6233.05  0.00  178318.00 | 4682.76  1758.78  8734.69  0.00  147901.20 | 3943.58  1486.95  7687.40  0.00  178318.00 | 3519.43  1456.89  6887.73  0.00  216920.88 | 4589.21  1914.12  9051.49  0.45  840992.57 | 4197.14  1720.75  8339.95  0.45  840992.57 |

All costs in €. Costs for screening are included. SCS = skin cancer screening; SD = standard deviation.
