# Supplementary material for: One-year follow-up healthcare costs of patients diagnosed with skin cancer in Germany: a claims data analysis
Source: BMC Health Serv Res. 2022 Jun 11;22:771. doi: 10.1186/s12913-022-08141-9 (PMC9188701; doi:10.1186/s12913-022-08141-9)
Supplement: Supplementary file 4 — Additional file 4. [file 12913_2022_8141_MOESM4_ESM.docx]

**Supplementary Table 4.** Results of the additional analyses.

| Parameter | Estimate | Standard error | 95% CI | | Z | Pr > \|Z\| |
| --- | --- | --- | --- | --- | --- | --- |
| **C43, without persons who died in the observation period** | | | | | | |
| Intercept | 7.4373 | 0.0327 | 7.3732 | 7.5014 | 227.58 | <.0001 |
| Scr | -0.2795 | 0.0316 | -0.3414 | -0.2175 | -8.84 | <.0001 |
| Per | 0.8105 | 0.0346 | 0.7426 | 0.8783 | 23.42 | <.0001 |
| Scr*Per | -0.1629^†^ | 0.0487 | -0.2582^†^ | -0.0675^†^ | -3.35 | 0.0008 |
| EH | 0.1001 | 0.0059 | 0.0884 | 0.1117 | 16.88 | <.0001 |
| PBM | 0.1385 | 0.0076 | 0.1235 | 0.1534 | 18.16 | <.0001 |
| **C44, without persons who died in the observation period** | | | | | | |
| Intercept | 7.2713 | 0.0138 | 7.2442 | 7.2984 | 525.37 | <.0001 |
| Scr | -0.1734 | 0.0131 | -0.1991 | -0.1477 | -13.20 | <.0001 |
| Per | 0.3338 | 0.0079 | 0.3184 | 0.3493 | 42.38 | <.0001 |
| Scr*Per | 0.0098^†^ | 0.0129 | -0.0155^†^ | 0.0351^†^ | 0.76 | 0.4481 |
| EH | 0.0974 | 0.0027 | 0.0921 | 0.1027 | 35.88 | <.0001 |
| PBM | 0.1661 | 0.0032 | 0.1598 | 0.1725 | 51.36 | <.0001 |
| **C43, from 35 to 64 years of age** | | | | | | |
| Intercept | 7.3259 | 0.0435 | 7.2406 | 7.4112 | 168.30 | <.0001 |
| Scr | -0.3289 | 0.0514 | -0.4296 | -0.2281 | -6.40 | <.0001 |
| Per | 0.9579 | 0.0519 | 0.8561 | 1.0597 | 18.45 | <.0001 |
| Scr*Per | -0.3290^†^ | 0.0697 | -0.4656^†^ | -0.1923^†^ | -4.72 | <.0001 |
| EH | 0.1613 | 0.0112 | 0.1395 | 0.1832 | 14.46 | <.0001 |
| PBM | 0.1614 | 0.0163 | 0.1295 | 0.1933 | 9.92 | <.0001 |
| **C44, from 35 to 64 years of age** | | | | | | |
| Intercept | 7.1518 | 0.0245 | 7.1038 | 7.1999 | 291.66 | <.0001 |
| Scr | -0.2998 | 0.0265 | -0.3517 | -0.2478 | -11.31 | <.0001 |
| Per | 0.3893 | 0.0217 | 0.3467 | 0.4318 | 17.95 | <.0001 |
| Scr*Per | -0.0272^†^ | 0.0277 | -0.0814^†^ | 0.0270^†^ | -0.98 | 0.3258 |
| EH | 0.1715 | 0.0072 | 0.1573 | 0.1856 | 23.72 | <.0001 |
| PBM | 0.2021 | 0.0102 | 0.1821 | 0.2220 | 19.86 | <.0001 |
| **C43, from 65 years of age onwards** | | | | | | |
| Intercept | 7.7200 | 0.0464 | 7.6291 | 7.8109 | 166.47 | <.0001 |
| Scr | -0.3538 | 0.0377 | -0.4277 | -0.2799 | -9.39 | <.0001 |
| Per | 0.7442 | 0.0384 | 0.6690 | 0.8194 | 19.40 | <.0001 |
| Scr*Per | -0.0436^†^ | 0.0587 | -0.1586^†^ | 0.0714^†^ | -0.74 | 0.4572 |
| EH | 0.0736 | 0.0063 | 0.0613 | 0.0858 | 11.76 | <.0001 |
| PBM | 0.1244 | 0.0095 | 0.1059 | 0.1429 | 13.16 | <.0001 |
| **C44, from 65 years of age onwards** | | | | | | |
| Intercept | 7.3736 | 0.0157 | 7.3428 | 7.4045 | 468.94 | <.0001 |
| Scr | -0.1770 | 0.0143 | -0.2051 | -0.1489 | -12.34 | <.0001 |
| Per | 0.3369 | 0.0092 | 0.3188 | 0.3550 | 36.50 | <.0001 |
| Scr*Per | 0.0426^†^ | 0.0151 | 0.0130^†^ | 0.0723^†^ | 2.82 | 0.0048 |
| EH | 0.0877 | 0.0021 | 0.0835 | 0.0919 | 40.81 | <.0001 |
| PBM | 0.1553 | 0.0029 | 0.1496 | 0.1609 | 53.81 | <.0001 |
| **C43, without costs for screening of individual person undergoing routine SCS*** | | | | | | |
| Intercept | 7.5284 | 0.0336 | 7.4625 | 7.5942 | 224.06 | <.0001 |
| Scr | -0.3606 | 0.0318 | -0.4228 | -0.2983 | -11.35 | <.0001 |
| Per | 0.8360 | 0.0317 | 0.7740 | 0.8981 | 26.39 | <.0001 |
| Scr*Per | -0.1696^†^ | 0.0467 | -0.2611^†^ | -0.0782^†^ | -3.63 | 0.0003 |
| EH | 0.1035 | 0.0061 | 0.0916 | 0.1155 | 16.99 | <.0001 |
| PBM | 0.1392 | 0.0082 | 0.1232 | 0.1552 | 17.03 | <.0001 |
| **C44, without costs for screening of individual person undergoing routine SCS*** | | | | | | |
| Intercept | 7.3214 | 0.0142 | 7.2935 | 7.3493 | 513.90 | <.0001 |
| Scr | -0.2157 | 0.0133 | -0.2417 | -0.1897 | -16.27 | <.0001 |
| Per | 0.3477 | 0.0085 | 0.3311 | 0.3644 | 40.96 | <.0001 |
| Scr*Per | 0.0276^†^ | 0.0131 | 0.0020^†^ | 0.0532^†^ | 2.11 | 0.0345 |
| EH | 0.1014 | 0.0027 | 0.0961 | 0.1066 | 37.96 | <.0001 |
| PBM | 0.1642 | 0.0031 | 0.1581 | 0.1703 | 52.61 | <.0001 |

CI = Confidence interval; Scr = dummy-variable to distinguish between routine SCS that has taken place and no routine SCS; Per = dummy-variable to distinguish between pre- and post-observation period; PBM = Pharmacy-based Metric; EH = Elixhauser Comorbidity Index; Screening*Period denotes the DiD estimator. †: Differences in coefficients between table and text arise as the text reports exact numbers, calculated as ((e^coefficient)-1)*100%. *: Screening costs were deducted pro rata in the pre and post period depending on the period in which they were incurred.
